# One of the Vagaries of the Anti-Vaccinationists

**Published:** 1882-12

**Authors:** 


### ONE OF THE VAGARIES OF THE ANTI-VACCINATIONISTS.

Every advance in the means of preventing disease meets more or less opposition from those whom it was designed to benefit. As it was in the days of Noah so it is now. Men will not get into the ark at the warning of the wise. They had rather have their own way and be destroyed than accept the views promulgated by others. In a late issue we called attention to the death of a learned physician from small-pox, far from home, because he would not be vaccinated. Such deaths are not at all unusual among the laity, but they are somewhat rare among educated medical men.

A pamphlet lies before us written by a Connecticut physician. In it the effort is made to class vaccination with Pasteur's alleged discoveries in disease prevention by means of the previous introduction of germs. Because Pasteur's conclusions are still unproved, our New England doctor tries to show that vaccination and Jenner are humbugs. As a fact the evidence for or against vaccination rests solely upon clinical experience. Has it or has it not hundreds of times demonstrated its power to protect a community from small-pox? This is a question of fact to be answered as any other question of fact.

To our mind the evidence that it has protected and that it does protect is as strong as the evidence in support of the revolution of the earth about the sun. We are told that some people do not believe this. Certainly we can believe that a mind which is so constituted as to reject the proofs in favor of vaccination, will also deny that the sun is the centre of the earth's motion. Science has not as yet demonstrated the morphological state of vaccine matter. If it is a germ, we have not seen the proof. If it be simply a peculiar form of excretion we have not seen an account of its composition. In short, we know nothing of its real nature, any more than we know the real nature of small-pox itself. We know something of the phenomena following its introduction into the human system, but of its real nature we are utterly ignorant. Hence it is not to be classed with Pasteur's researches concerning the nature and prophylaxis of charbon. Pasteur claimed to have discovered that a definite organism produced charbon in sheep, and that another definite organism would destroy that of charbon in some manner perhaps, as ducks eat earth worms. Hence he reasoned that if the destroyers of the charbon germs were introduced into the sheep before the charbon germs, they would protect the sheep by eating up or otherwise rendering harmless the deadly germs. Extended experiments have thus far failed to establish these claims. Hence, at the present time Pasteur's researches count for but little.

But what we object to in the pamphlet before us and in all similar writings, is the claim that because these researches of Pasteur lack a rational basis, therefore vaccination lacks a rational basis. The evidence upon which vaccination calls for the attention of all intelligent persons is totally different from that adduced in support of Pasteur's work. We are passing through a craze respecting the causation of disease. This craze is the idea that germs produce most forms of disease. Minute organism do produce itch, and a considerable number of cutaneous lesions, and some internal or constitutional affections as trichinosis, etc. But that the exanthematous diseases are produced by germs is certainly not proved, and all talk about this or about the germs of vaccine matter is speculation only. Any effort to ally vaccination with Pasteur's researches respecting charbon, etc., is unscientific and misleading. Better let vaccination stand on its solid basis of clinical demonstration, just as small-pox stands.—*Detroit Lancet.*

